# Innovative endoscopic combination surgery for endoscopic submucosal dissection using two thin therapeutic endoscopes

**DOI:** 10.1055/a-2299-2534

**Published:** 2024-05-29

**Authors:** Takuma Okamura, Tomonari Ikeda, Kazuyoshi Nagata, Tatsuki Ichikawa, Kazuhiko Nakao

**Affiliations:** 1 13650 Department of Gastroenterology, Nagasaki Harbor Medical Center, Nagasaki, Japan; 2 200674 Department of Comprehensive Community Care Systems, Nagasaki University School of Medicine Graduate School of Biomedical Sciences, Nagasaki, Japan; 3 88380 Department of Gastroenterology, Nagasaki University Hospital, Nagasaki, Japan

Endoscopic procedures are complicated and difficult to perform because they are performed using a single endoscope. Although double-scope endoscopic submucosal dissection (ESD) with an ultrathin endoscope and a normal-diameter endoscope or two normal-diameter endoscopes has been used 1 , problems arise, such as reduced operability due to interference between the scope and the limited instruments that can be used. Recently, a narrow endoscope (EG-840TP, FUJIFILM, Tokyo, Japan) was developed and reported to be useful for endoscopic resection 2 3 with a relatively narrow 7.9-mm outer diameter and a large 3.2-mm accessory channel. We devised a thin endoscopic combination surgery that uses two thin therapeutic scopes to reduce the effects of mutual interference and allow free movement. This allows the two instruments to be moved independently and simultaneously, as in laparoscopic surgery, or observed and assisted by an assistive scope that does not perform the procedure, to ensure that the procedure is targeted to the appropriate location. Here, we performed endoscopic submucosal dissection of a large rectal tumor using thin endoscopic combination surgery ( Fig. 1 , Fig. 2 ; Video 1 ). The operator performed the resection with a knife, while the assistant assisted with local injection, real-time traction to the required area using grasping forceps, and aspiration of accumulated water ( Fig. 3 ). Because the field of view can be freely developed, we do not need to dive into the submucosa or attach the tip. We achieved en bloc resection within 159 min without any adverse events. After resection, most large tumors are difficult to retrieve; however, the tumor can be easily stored in an endoscopic retrieval bag (ENDO CARRY, Hakko, Tokyo, Japan) as a laparoscope using thin endoscopic combination surgery, and the resected specimen can be retrieved without damage ( Fig. 4 ).

**Fig. 1:**
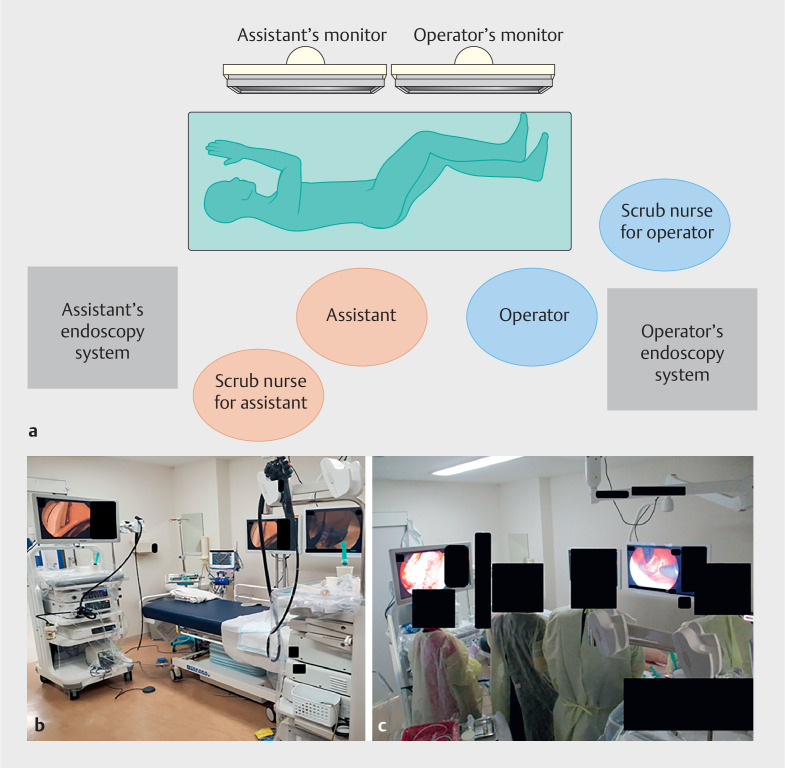
**a** Schematic of the layout of the endoscopic operating room. **b** Layout of the endoscopic operating room. **c** Positions of the operator and scrub nurse during the procedure (from left to right: scrub nurse for the assistant, assistant, operator, and scrub nurse for the operator).

**Fig. 2:**
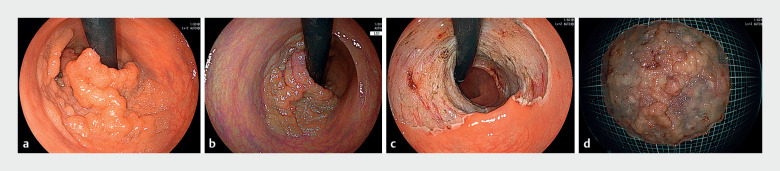
Endoscopic view of the rectal tumor. **a** White light image of a large rectal tumor. **b** Linked color imaging of the tumor. **c** After endoscopic resection. **d** The resected specimen measured 112 × 104 mm, with a lesion size of 102 × 98 mm.

**Fig. 3:**
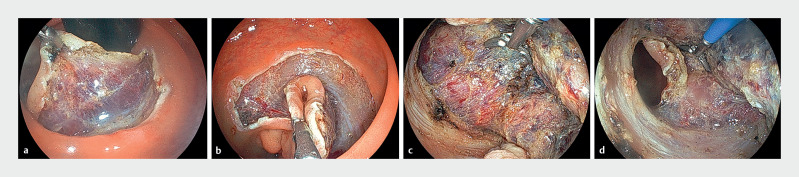
Endoscopic view of submucosal dissection with thin endoscopic combination surgery. **a** When the submucosa is entered, a good field of view can be immediately created by traction using the grasping forceps. **b** The view of the area to be resected can always be secured using real-time traction by changing the gripping point and traction direction, even in areas that are difficult to see in the lateral direction. **c, d** During submucosal dissection, the field of view can be secured while pushing up with the forceps.

**Fig. 4:**
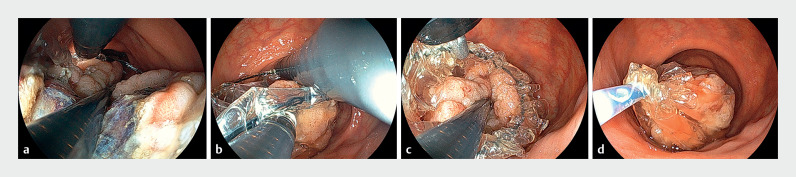
Endoscopic view of retrieval with thin endoscopic combination surgery. **a** Large tumor in the retrieval bag using thin endoscopic combination surgery. **b** The assistant lifts the bag edge, and the operator lifts the front edge and closes the bag. **c** The tumor was pushed into the bag by the operator as appropriate. **d** The tumor was stored completely inside the bag.

Innovative thin endoscopic combination surgery for endoscopic procedures.Video 1

Thin endoscopic combination surgery is useful for both ESD and specimen retrieval and may be applied to various endoscopic procedures.

Endoscopy_UCTN_Code_TTT_1AQ_2AD_3AD

## Notes

Endoscopy E-Videos https://eref.thieme.de/e-videos
*E-Videos* is an open access online section of the journal *Endoscopy* , reporting on interesting cases and new techniques in gastroenterological endoscopy. All papers include a high-quality video and are published with a Creative Commons CC-BY license. Endoscopy E-Videos qualify for HINARI discounts and waivers and eligibility is automatically checked during the submission process. We grant 100% waivers to articles whose corresponding authors are based in Group A countries and 50% waivers to those who are based in Group B countries as classified by Research4Life (see: https://www.research4life.org/access/eligibility/ ). This section has its own submission website at https://mc.manuscriptcentral.com/e-videos .
